# Elucidating Distinct and Common fMRI-Complexity Patterns in Preadolescent Children With Attention-Deficit/Hyperactivity Disorder, Oppositional Defiant Disorder, and Obsessive-Compulsive Disorder

**DOI:** 10.1016/j.jaacop.2025.11.008

**Published:** 2025-11-27

**Authors:** Ru Zhang, Steven Cen, Dilmini Wijesinghe, Leon Aksman, Stuart B. Murray, Christina J. Duval, Danny J.J. Wang, Kay Jann

**Affiliations:** a University of California San Francisco, San Francisco, California; b University of Southern California, Los Angeles, California; c University of California, Los Angeles, Los Angeles, California; d St. Louis University, St. Louis, Missouri

**Keywords:** attention-deficit/hyperactivity disorder (ADHD), oppositional defiant disorder (ODD), obsessive-compulsive disorder (OCD), resting-state fMRI, sample entropy

## Abstract

**Objective:**

The pathophysiology of attention-deficit/hyperactivity disorder (ADHD) is complicated by high rates of psychiatric comorbidities; thus, delineating unique vs shared functional brain perturbations is critical in elucidating illness pathophysiology. We investigated resting-state functional magnetic resonance imaging (rsfMRI)–complexity alterations among children with ADHD, oppositional defiant disorder (ODD), and obsessive-compulsive disorder (OCD), respectively, and comorbid ADHD, ODD, and OCD, within the cool and hot executive function (EF) networks.

**Method:**

We leveraged baseline data from 9- to 10-year-old children in the Adolescent Brain and Cognitive Development (ABCD) Study. Data for children who singularly met all *DSM-5* behavioral criteria for ADHD (n = 61), ODD (n = 38), and OCD (n = 48), respectively, were extracted, alongside data for children with comorbid ADHD, ODD, OCD, and/or other psychiatric diagnoses (n = 833). Data for a control sample of age-, sex-, and developmentally matched children were also extracted (N = 269). Voxel-wise sample entropy (SampEn) was computed using the LOFT Complexity Toolbox. Mean SampEn within all regions of interest (ROIs) of the EF networks was calculated for each participant. Hierarchical models with generalized estimating equations compared SampEn of comorbidity-free and comorbid ADHD, ODD, and OCD within the EF networks.

**Results:**

SampEn was reduced in comorbidity-free ADHD and ODD in overlapping regions of both EF networks compared with the healthy controls, including the bilateral superior frontal gyrus, anterior/posterior cingulate gyrus, and bilateral caudate (Wald statistic = 5.682-10.798, *p* < .05, and Benjamini–Hochberg [BH] corrected), with ADHD additionally affected in the right inferior/middle frontal gyrus and bilateral frontal orbital cortex (Wald statistic = 7.231-9.420, *p* < 0.05, and BH corrected). Among comorbid presentations, the presence of ADHD symptomatology was associated with significantly lower SampEn in every ROI (*z* = −3.973 to −2.235, *p* < .05, and BH corrected).

**Conclusion:**

ADHD and ODD shared common impairments underlying the EF networks in the comorbidity-free presentations, with ADHD showing more widespread complexity reduction. When ADHD co-occurred with other psychiatric disorders, the reduction in SampEn extended beyond the regions affected in comorbidity-free ADHD, indicating that comorbidities amplify neural complexity deficits. In contrast, no significant SampEn alterations were observed in OCD, whether presented alone or in combination with ADHD.

## Plain language summary

Attention-deficit/hyperactivity disorder (ADHD) and its comorbidities may be better understood by examining differences in the brains of those with ADHD versus ADHD with other psychiatric diagnoses. Using data from the Adolescent Brain and Cognitive Development℠ (ABCD) study, this study compares the resting-state fMRI executive function networks in 9- to 10-year-old children. Researchers found ADHD and oppositional defiant disorder (ODD) shared common impairments in the comorbid-free presentations, with ADHD showing more widespread reductions in functional brain complexity. When ADHD co-occurred with other psychiatric disorders, the reduction in complexity extended beyond the regions affected in comorbid-free ADHD, indicating that comorbidities amplify deficits in neural complexity. In contrast, no significant complexity alterations were observed in obsessive-compulsive disorder (OCD), whether presented alone or in combination with ADHD.

Attention-deficit/hyperactivity disorder (ADHD) is a childhood neurodevelopmental disorder that is defined by developmentally inappropriate levels of inattention and/or hyperactivity-impulsivity.^1^ ADHD is estimated to affect 11.3% of children and adolescents and approximately 4.4% of adults in the United States.^2^^,^^3^ Although numerous studies have been conducted on ADHD, the pathophysiology of the disorder is still not fully understood, especially because of the high rate of comorbidities. Two commonly observed comorbid presentations are obsessive-compulsive disorder (OCD) and oppositional defiant disorder (ODD). ODD is defined by a frequent and persistent pattern of irritable and angry mood, vindictiveness, and developmentally inappropriate, negativistic, defiant, and disobedient behavior toward authority figures.^1^ OCD is usually depicted by the presence of recurrent, intrusive, and worrying thoughts, which often elicit repetitive behavior carried out with the aim of neutralizing negative feelings caused by the obsessions.^1^ The comorbidity rate of ODD within children and adolescents with ADHD is around 26% and the rate of co-occurrence of OCD is reported to be 3% to 7.5%.^4^^,^^5^

All 3 disorders are characterized by deficits in executive function (EF). EF can be distinguished into cool and hot EF.^6^ Cool EF refers to goal-directed and problem-solving behaviors, as well as self-regulation, not involving motivational or affective aspects. Cool EF encompasses functions using diverse abilities such as inhibition, working memory, planning, flexibility, and the ability to creatively generate solutions for problems. Brain areas that are central to cool EF include the lateral prefrontal cortex, dorsal anterior cingulate cortex, and hippocampus. In contrast, hot EF is characterized by motivational and affective aspects of cognitive processing, such as reinforcement learning, affective decision making, and emotional processing. Brain areas putatively implicated in hot EF include the amygdala, nucleus accumbens, ventral striatum, ventral anterior cingulate cortex, ventromedial prefrontal cortex, orbitofrontal cortex, and posterior cingulate cortex. ADHD is characterized predominantly by structural and functional brain alterations in the cool EF system, whereas ODD is associated with abnormalities in the hot EF system, and OCD may include alterations in both.^7^, ^8^, ^9^

Nonlinear analyses from functional magnetic resonance imaging (fMRI) that characterize neural signal complexity have been proposed as measures for the information-processing capacity of brain areas and networks^10^, ^11^, ^12^, ^13^ or indices of pathological brain function.^14^, ^15^, ^16^ Sample entropy (SampEn) is a nonlinear, model-free measure of signal complexity that quantifies the irregularity and unpredictability of time-series data. It estimates the likelihood that similar sequences of data points remain similar at the next point in time, within a defined tolerance. Although metrics such as amplitude of low-frequency fluctuations (ALFF) or standard deviation of the neural signal measure the magnitude or power of fluctuations,^17^, ^18^, ^19^, ^20^, ^21^, ^22^, ^23^ they do not account for the temporal ordering or structure of the signal. SampEn, on the other hand, assesses how predictable patterns of signal values are over time, thus capturing dynamic features of the neural signal that reflect functional brain complexity and self-regulation. Higher entropy generally reflects more irregular and information-rich neural signal fluctuations, which may indicate greater local processing capacity. Conversely, lower entropy suggests more regular, predictable signal patterns, which can reflect reduced neural variability and potentially diminished adaptability. Several studies have reported that brain entropy increases from childhood through adolescence, reaches its peak in adulthood, and then gradually declines during aging.^24^^,^^25^ Brain complexity has also been linked to measures of intelligence and cognitive performance, although the direction of this association remains uncertain.^24^^,^^26^^,^^27^

Traditional functional connectivity approaches measure the statistical dependence between spatially distinct brain regions, most commonly using correlations of neural time series.^28^ These methods have been invaluable for mapping large-scale networks and identifying disrupted communication across regions in disease states. However, they primarily capture inter-regional synchrony and overlook the temporal dynamics within each local signal. SampEn characterizes the complexity of local signals themselves, capturing the intrinsic dynamics within individual regions. It does not replace conventional connectivity metrics but instead offers a complementary perspective on brain function. SampEn has been used to detect functional brain alterations in clinical populations, for instance individuals with ADHD. A few studies have investigated the complexity of resting-state fMRI (rsfMRI) data in ADHD, 2 for adult ADHD^29^^,^^30^ and 1 study for preadolescent ADHD,^31^ and all have reported reduced complexity in the frontal cortex. The complexity studies in OCD have been focused on adult participants, and the results are diverse. Only 1 study used the modality of rsfMRI, and the results revealed no significant difference in SampEn between individuals with OCD and controls.^32^ One electroencephalography study indicated lower complexity in individuals with OCD, with the affected areas including the prefrontal and frontotemporal cortices^33^; another study reported higher complexity in individuals with OCD in the frontal regions.^34^ No study has investigated the complexity of ODD.

Our prior study^31^ investigated differences in rsfMRI complexity between children with comorbidity-free ADHD and age-, sex-, and youth pubertal development status (YPDS)–matched healthy controls using data from the Adolescent Brain Cognitive Development℠ (ABCD) Study. Although this study provided important initial evidence for altered brain signal complexity in comorbidity-free ADHD, it did not address diagnostic comorbidity or focus on specific functional networks linked to executive function. In addition, investigating ADHD alongside ODD and OCD is crucial, as these conditions frequently co-occur, yet their neural interactions remain poorly understood. The current study represents the first attempt to explore functional brain alterations in ADHD, ODD, and OCD, both in comorbidity-free and comorbid presentations. We examined SampEn of ADHD, ODD, and OCD in the cool and hot EF networks when they were comorbidity-free disorders, as well as when these symptomatologies co-occurred. We hypothesized the following: (1) SampEn for comorbidity-free ADHD, ODD, and OCD would be lower when compared to values in healthy controls; and (2) SampEn in the presence of ADHD, ODD, and OCD symptomatologies would be lower than those in absence. By integrating comorbidity distinctions and network-level specificity, the current study provides a more clinically grounded and mechanistically targeted characterization of functional brain alterations than previously available in the literature.

## Method

### Participants and Procedures

We leveraged the baseline demographic, clinical, T1 structural, and rsfMRI data from release 3.0 of ABCD Study®.^35^ The ABCD baseline included 11,878 participants in total, and each was evaluated based on 144 psychiatric diagnoses (Figure S1, available online). In all, 1,085 of the participants were diagnosed with ADHD. In release 3.0, ADHD diagnoses were made according to *DSM-5* criteria,^1^ according to parent reports by the Kiddie-Schedule for Affective Disorders and Schizophrenia (KSADS)–Parent Report. A total of 1,085 baseline participants were diagnosed with ADHD with or without various comorbidities, comprising 9.1% of the entire ABCD baseline sample. This aligns with previous studies, which have reported that ADHD affects approximately 11.3% of children and adolescents in the United States.^2^ However, because of an anomaly within the ABCD Study infrastructure, what were initially confirmed as ADHD diagnoses were later determined to be invalid, as parents were asked only whether “symptoms interfere with social, academic, or occupational functioning.” Full *DSM-5* criteria require that impairment extends to at least 2 domains. Consequently, full-threshold *DSM-5* diagnoses could not be confirmed. Our sample therefore comprised children who met all behavioral criteria for ADHD but without confirmation that impairment occurred across multiple domains. In this sense, the sample reflected children with all behavioral features of ADHD.

We divided the whole ADHD sample into 8 groups, as follows: (1) ADHD, (2) ADHD with comorbid ODD, (3) ADHD with comorbid OCD, (4) ADHD with comorbid ODD and OCD, (5) ADHD with another psychiatric comorbidity, (6) ADHD with comorbid ODD and another psychiatric comorbidity, (7) ADHD with comorbid OCD and another psychiatric comorbidity, and (8) ADHD with comorbid ODD, OCD, and another psychiatric comorbidity.

In addition, we selected these groups of comorbidity-free ODD and comorbidity-free OCD. We also pseudorandomly selected a healthy control group. Specifically, we first stratified the full ADHD sample (N = 1,085) by age range, YPDS, and sex, recording the number of individuals in each subgroup. We then filtered the healthy participant pool to identify individuals who matched each of these subgroups, and we randomly selected an equal number of controls accordingly. These selected healthy controls were combined into a matched control pool, and we confirmed that the overall distributions of age, sex, and YPDS were comparable to those of the full ADHD sample, with no significant differences (*p* > .05). From this matched pool of 1,085 healthy controls, 300 individuals were randomly selected, and this subsample remained well matched to the full ADHD group in terms of age, sex, and YPDS (*p* > .05).

We additionally required each participant to have T1 structural data and at least 2 complete rsfMRI scans. Participants with mean framewise displacement (FD) of >0.2 mm in any rsfMRI scan run were excluded. In the final sample, participants with comorbidity-free ADHD, ODD, and OCD were matched with healthy controls in terms of sex, age, YPDS, and average of FD (avgFD) over 2 rsfMRI scan runs, and scan site (Table 1^36^, ^37^, ^38^). The other ADHD groups were matched with the healthy controls in terms of sex, age, and YPDS but not avgFD and scan-site (*p* < .05) (Table 2).

**Table 1:** Overview of Sample Characteristics Delineated by Group for the Analysis of Comorbidity-Free Attention-Deficit/Hyperactivity Disorder, Oppositional Defiant Disorder, and Obsessive-Compulsive Disorder

Group	Sex, F/M	Age, y	YPDS	ADHD score	ODD score	OCD score	avgF, mm	Scan-site
Control	89/180	9.946 ± 0.629	1.607 ± 0.472	1.059 ± 1.561	0.784 ± 1.272	0.654 ± 1.104	0.025 ± 0.013	—
Comorbidity-free ADHD	24/37	9.907 ± 0.649	1.646 ± 0.538	5.443 ± 2.180	1.456 ± 1.699	1.213 ± 1.529	0.030 ± 0.023	—
Comorbidity-free ODD	16/22	9.969 ± 0.720	1.547 ± 0.365	1.526 ± 2.023	3.316 ± 1.210	0.711 ± 0.927	0.025 ± 0.011	—
Comorbidity-free OCD	14/34	9.993 ± 0.670	1.503 ± 0.378	1.458 ± 1.786	1.208 ± 1.515	1.667 ± 1.464	0.030 ± 0.018	—
Group comparison	*χ**^2^**d* = 0.019, *p* = .890	*F* = 0.174, *p* = .914	*F* = 1.066, *p* = .363	*F* = 107, *p* = .000∗	*F* = 39.32, *p* = .000∗	*F* = 11.7, *p* = .000∗	*F* = 2.314, *p* = .075	*χ**^2^**d* = 79.596, *p* = .084

Note: The ADHD, ODD, and OCD scores were measured by the parent-reported ADHD CBCL-DSM5 scale, Opposit CBCL DSM5 Scale, and Obsessive-Compulsive Problems (OCP) CBCL 2007 Scale, respectively.^36^^,^^37^ ADHD = attention-deficit/hyperactivity disorder; AvgFD = average FD over 2 rsfMRI scan runs; FD = framewise displacement; OCD = obsessive-compulsive disorder; ODD = oppositional defiant disorder; YPDS = youth pubertal development status, measured by the self-reported pubertal development scale.^38^

∗ *p* < .05.

**Table 2:** Overview of Sample Characteristics Delineated by Group for the Analysis of Comorbid Attention-Deficit/Hyperactivity Disorder (ADHD), Oppositional Defiant Disorder (ODD), and Obsessive-Compulsive Disorder

Group	Sex, F/M	Age, y	YPDS	ADHD score	ODD score	OCD score	AvgF, mm	Scan site
Control	89/180	9.946 ± 0.629	1.607 ± 0.472	1.059 ± 1.561	0.784 ± 1.272	0.654 ± 1.104	0.025 ± 0.013	—
ADHD+ODD+X	49/136	9.920 ± 0.632	1.711 ± 0.539	8.632 ± 3.055	6.173 ± 2.135	2.973 ± 2.525	0.030 ± 0.018	—
ADHD+OCD+X	37/82	9.838 ± 0.604	1.653 ± 0.478	7.840 ± 2.668	3.613 ± 2.322	4.496 ± 2.699	0.033 ± 0.022	—
ADHD+ODD+OCD+X	24/53	9.937 ± 0.646	1.658 ± 0.478	9.364 ± 2.901	6.481 ± 2.251	5.442 ± 3.114	0.026 ± 0.016	—
ADHD+X	148/294	9.906 ± 0.617	1.684 ± 0.516	7.434 ± 2.755	3.052 ± 2.179	2.385 ± 2.227	0.030 ± 0.020	—
ADHD+ODD	2/3	9.567 ± 0.308	1.390 ± 0.459	5.400 ± 2.302	4.000 ± 1.414	1.200 ± 2.683	0.028 ± 0.012	—
ADHD+OCD	1/3	9.958 ± 0.685	1.600 ± 0.432	6.750 ± 4.272	2.750 ± 2.217	2.500 ± 1.915	0.040 ± 0.016	—
ADHD+ODD+OCD	1/0	10.333	2.600	4.000	9.000	4.000	0.046	—
Group comparison	*χ**^2^* = 5.612, *p* = .586	*F* = 0.674, *p* = .695	*F* = 1.555, *p* = .145	*F* = 214.4, *p* = .000∗	*F* = 144.7, *p* = .000∗	*F* = 62.09, *p* = .000∗	*F* = 3.179, *p* = .002∗	*χ**^2^* = 1.555, *p* = .030∗

Note: The healthy controls in Table 2 were identical to those in Table 1. The ADHD, ODD, and OCD scores were measured by the parent-reported ADHD CBCL-DSM5 scale, Opposit CBCL DSM5 Scale, and Obsessive-Compulsive Problems (OCP) CBCL 2007 Scale, respectively.^36^^,^^37^ ADHD = attention-deficit/hyperactivity disorder; ADHD+OCD = participants with ADHD and OCD; ADHD+OCD+X = participants with ADHD, OCD, and X; ADHD+ODD = participants with ADHD and ODD; ADHD+ODD+OCD = participants with ADHD, ODD, and OCD; ADHD+ODD+OCD+X = participants with ADHD, ODD, OCD, and X; ADHD+ODD+X = participants with ADHD, ODD, and X; ADHD+X = participants with ADHD and X; AvgFD = average FD over 2 rsfMRI scan runs; OCD = obsessive-compulsive disorder; ODD = oppositional defiant disorder; FD = framewise displacement; X = participants with 1 or more other psychiatric diagnoses, excluding ADHD, ODD, and OCD. YPDS = youth pubertal development status, measured by the self-reported pubertal development scale.^38^

∗ *p* < .05.

### Data Analyses

#### Imaging Acquisition and Preprocessing

All T1-weighted scans were acquired with voxel resolution = 1 mm^3^, 256 × 256 matrix, flip angle = 8°, and 2× parallel imaging. Other scan parameters slightly varied by scanner platform, namely, Siemens Prisma, Philips, or GE 3T scanner. Each participant had 3 to 4 eyes-open (passive crosshair viewing) rsfMRI scans, each of which was approximately 5 minutes in duration. All rsfMRI scans were collected using a gradient-echo EPI sequence of 383 volumes in total (voxel resolution = 2.4 × 2.4 × 2.4 mm^3^, 60 slices, 90 × 90 matrix, field of view = 216 × 216, TR = 800 milliseconds, TE = 30 milliseconds, flip angle = 52°, 6-factor multiband acceleration).

For the current study, we used rsfMRI run 1 and run 2. For each participant, the T1 structural and resting-state fMRI data were preprocessed using the CONN toolbox (CONN: fMRI functional connectivity toolbox). The pipeline included the following steps: (1) functional realignment and unwarping, which corrected for head motion and susceptibility-related distortions by aligning all functional volumes to the first volume; (2) slice-timing correction to adjust for differences in slice acquisition timing; (3) structural segmentation and normalization, where the T1-weighted anatomical image was segmented into tissue classes and normalized to Montreal Neurological Institute (MNI) space using nonlinear transformations; (4) functional normalization, applying the same transformation to functional images, resampled to 2-mm isotropic voxels; and (5) spatial smoothing with a 6-mm full-width at half-maximum (FWHM) Gaussian kernel.

### Complexity Analysis

SampEn is defined as the natural logarithm of the conditional probability that a pattern length of *m* points will repeat itself, excluding self-matches, for *m* + 1 points within a tolerance of *r* in a time series of length *N.*^39^ We set the pattern length *m* = 2 and the sensitivity threshold *r* = 0.3 in the current study.^40^ For each preprocessed rsfMRI time series, the first 5 volumes were removed, and no additional volumes were scrubbed. SampEn was then computed voxel-wise using the LOFT Complexity Toolbox and averaged across the 2 rsfMRI runs for each participant.

Mean SampEn of each region-of-interest (ROI) of the 2 networks was calculated for each participant: the EF networks were defined based on the recommendations of Salehinejad *et al.*^6^ Specifically, the ROIs selected for each network matched those illustrated in Figure 4 of their review. Our ROIs based on the Harvard–Oxford atlas^41^ for the cool EF network included the superior frontal gyrus, middle frontal gyrus, inferior frontal gyrus, anterior cingulate cortex, and hippocampus.^6^ The hot EF network included the amygdala, accumbens, caudate, putamen, anterior cingulate cortex, frontal medial cortex, frontal orbital cortex, and posterior cingulate gyrus. The Harvard–Oxford atlas does not distinguish between the dorsal and ventral subdivisions of the anterior cingulate cortex. Therefore, we used the entire anterior cingulate cortex as a single ROI for both EF networks. The mean SampEn of each network was also calculated for each participant.

#### Analysis for Comorbidity-Free ADHD, ODD, and OCD

Hierarchical models with generalized estimating equations (GEE) were implemented to investigate SampEn of the participants with comorbidity-free ADHD, ODD, and OCD vs healthy controls using avgFD as a covariate and scan-site as a clustering variable for (1) each EF network as a whole and (2) each ROI in each EF network separately (information on the participants used in this analysis in Table 1):

$${\text{SampEn}}_{sj}=\beta_{0}+\beta_{1}I\left\{{\text{comorbidity-free}\;\text{ADHD}}_{sj}\right\}+\beta_{2}I\left\{{\text{comorbidity-free}\;\text{ODD}}_{sj}\right\}+\beta_{3}I\left\{{\text{comorbidity-free}\;\text{OCD}}_{sj}\right\}+\beta_{4}\;{\text{avgFD}}_{sj}\text{,}$$

where I was the indicator variable, in which 1 = comorbidity-free disorder present and 0 = comorbidity-free disorder absent, *s* = 1,…, *S* denoted the clustering factor scan-site, and *j* = 1, …, *n*_*s*_ denoted the participant. Benjamini–Hochberg (BH) correction^42^ with a false discovery rate of *q* < 0.05 was performed for all the network-wise and ROI analyses in this article.

### Analysis for the Whole ADHD Sample

Hierarchical models with GEE were implemented to investigate statistical differences in SampEn of the whole ADHD sample vs. healthy controls using avgFD as a covariate and scan-site as the clustering factor for (1) each EF network and (2) each ROI in each EF network:

$${\text{SampEn}}_{sj}=\beta_{0}+\beta_{1}I\left\{{\text{ADHD}}_{sj}\right\}+\beta_{2}\;{\text{avgFD}}_{sj}\text{,}$$

where I was the indicator variable, in which 1 = ADHD present and 0 = healthy control, *s* = 1,…, *S* denoted the clustering factor scan-site, and *j* = 1, …, *n*_*s*_ denoted the participant. Here, the whole ADHD sample included individuals with ADHD with all kinds of comorbidities displayed in Table 2 and comorbidity-free ADHD displayed in Table 1. It should be noted that the healthy controls in Table 2 were identical to those in Table 1.

### Analysis for Comorbid ADHD, ODD, and OCD

Hierarchical models with GEE were used to investigate SampEn affected by ADHD symptomatology (presence vs absence), ODD symptomatology (presence vs absence), and OCD symptomatology (presence vs absence) for (1) each EF network, and (2) each ROI in each EF network:

$${\text{SampEn}}_{sj}=\beta_{0}+\beta_{1}I\left\{{\text{ADHD}\;\text{symptomatology}}_{sj}\right\}+\beta_{2}I\left\{{\text{ODD}\;\text{symptomatology}}_{sj}\right\}+\beta_{3}I\left\{{\text{OCD}\;\text{symptomatology}}_{sj}\right\}+\beta_{4}I\left\{{X\;\text{symptomatology}}_{sj}\right\}+\beta_{5}\;{\text{avgFD}}_{sj}\text{.}$$

For the indicator variable I, 1 = symptomatology present and 0 = symptomatology absent. In this model, ADHD, ODD, and OCD symptomatologies were treated as the independent variables, X symptomatology and avgFD as covariates, scan-site *s* = 1,…, *S* as the clustering factor, and *j* = 1, …, *n*_*s*_ as the participant. Detailed information on the ADHD participants with comorbid presentations and healthy controls is displayed in Table 2; only those participants represented in this table were used in this analysis.

### Secondary Analyses

#### Adding Sociodemographic Covariates

We replicated the 3 main analyses by adding age, sex, and YPDS as additional covariates in the hierarchical models with GEE.

#### Splitting of the Other Comorbidities

We replicated the analysis for comorbid ADHD, ODD, and OCD by dividing the X symptomatology into 3 categories: mood disorder symptomatology, anxiety symptomatology, and other symptomatology (Supplement 1, available online, provides more details).

#### Effects of Number of Comorbidities

Hierarchical models with GEE were used to evaluate the potential association between SampEn and the number of comorbidities for (1) each EF network, and (2) each ROI in each EF network with avgFD as the covariate and scan-site *s* as the clustering factor:

$${\text{SampEn}}_{sj}=\beta_{0}+\beta_{1}{\text{Number}\;\text{of}\;\text{comorbidities}}_{sj}+\beta_{2}\;{\text{avgFD}}_{sj}\text{.}$$

The whole ADHD sample was included in this model, but all of the healthy controls were excluded.

### Association Between Complexity and Symptom Severity

The ADHD, ODD, and OCD scores were measured by the parent-reported ADHD CBCL-DSM5 scale, Opposit CBCL DSM5 Scale, and Obsessive-Compulsive Problems (OCP) CBCL 2007 Scale, respectively.^36^^,^^37^ Hierarchical models with GEE were used to evaluate the potential association between SampEn and the raw ADHD score within the whole ADHD sample for (1) each EF network, and (2) each ROI in each EF network with avgFD as the covariate and scan-site *s* as the clustering factor:

$${\text{SampEn}}_{sj}=\beta_{0}+\beta_{1}{\text{ADHD}\;\text{score}}_{sj}+\beta_{2}\;{\text{avgFD}}_{sj}\text{.}$$

The same analysis was replicated for the raw ODD score within the whole ODD sample and the raw OCD score within the whole OCD sample.

#### Medication Effects

Hierarchical models with GEE examined the main effect of medication (medicated vs unmedicated) within the whole ADHD sample for (1) each EF network, and (2) each ROI in each EF network with avgFD as the covariate and scan-site *s* as the clustering factor:

$${\text{SampEn}}_{sj}=\beta_{0}+\beta_{1}I\left\{{\text{Medication}}_{sj}\right\}+\beta_{2}\;{\text{avgFD}}_{sj}\text{.}$$

For the indicator variable I, 1 = medicated and 0 = unmedicated. Table S1, available online, provides the list of prescriptions. The ADHD participants who consumed at least 1 prescription were categorized as medicated.

## Results

### Clinical Results

There was no statistical difference in terms of sex (*χ*^2^= 0.019, *p* > .05), age (*F* = 0.174, *p* > .05), YPDS (*F* = 1.066, *p* > .05), avgFD (*F* = 2.314, *p* > .05), and scan-site (*χ*^2^= 79.596, *p* > .05) when comparing participants with comorbidity-free ADHD, ODD, and OCD and the healthy controls (Table 1). There was no statistical difference in terms of sex (*χ*^2^= 5.612, *p* > .05), age (*F* = 0.674, *p* > .05), and YPDS (*F* =1.555, *p* > .05), but not avgFD (*F* = 3.179, *p* < .05) and scan-site (*χ*^2^= 1.555, *p* < .05) when comparing the comorbid ADHD groups and the controls (Table 2).

### Complexity Analysis

**Figure 1:**
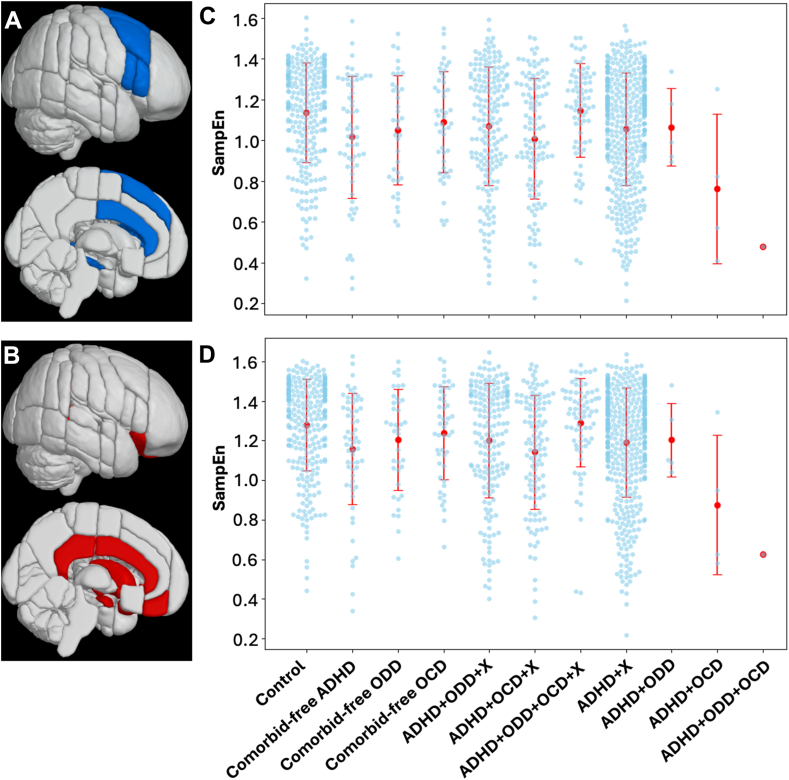
Mean Sample Entropy for Each Executive Function Network and Group ***Note:****(a) The cool EF network. (b) Mean SampEn of the cool EF network for each participant, plotted against each group. The red dots and line segments display the means and SDs for the groups. (c) The hot EF network. (d) Mean SampEn of the hot EF network for each participant, plotted against each group. The red dots and line segments display the means and SDs for the groups. Note: ADHD = attention-deficit/hyperactivity disorder; ADHD+OCD = participants with ADHD and OCD; ADHD+OCD+X = participants with ADHD, OCD, and X; ADHD+ODD = participants with ADHD and ODD; ADHD+ODD+OCD = participants with ADHD, ODD, and OCD; ADHD+ODD+OCD+X = participants with ADHD, ODD, OCD, and X; ADHD+ODD+X = participants with ADHD, ODD, and X; ADHD+X = participants with ADHD and X; EF = executive function; OCD = obsessive-compulsive disorder; ODD = oppositional defiant disorder; SampEn = sample entropy; SD = standard deviation; X = participants with 1 or more other psychiatric diagnoses, excluding ADHD, ODD, and OCD.*

Mean SampEn values for each participant, stratified by group, are shown for both the cool EF network and the hot EF network in Figure 1.

### Analysis for Comorbid-Free ADHD, ODD, and OCD

On the network level analysis, we found significant main effects of Group in each network (*p* < .05 and BH corrected). Post hoc tests indicated that participants with comorbidity-free ADHD and comorbidity-free ODD had significantly lower SampEn than the controls in the cool EF network (Wald statistic = 6.920 and 5.371, *p* < .05, and BH corrected) and in the hot EF network (Wald statistic = 8.640 and 5.577, *p* < .05, and BH corrected). SampEn of participants with comorbidity-free OCD was not significantly different from that of the controls in either EF network.

**Figure 2:**
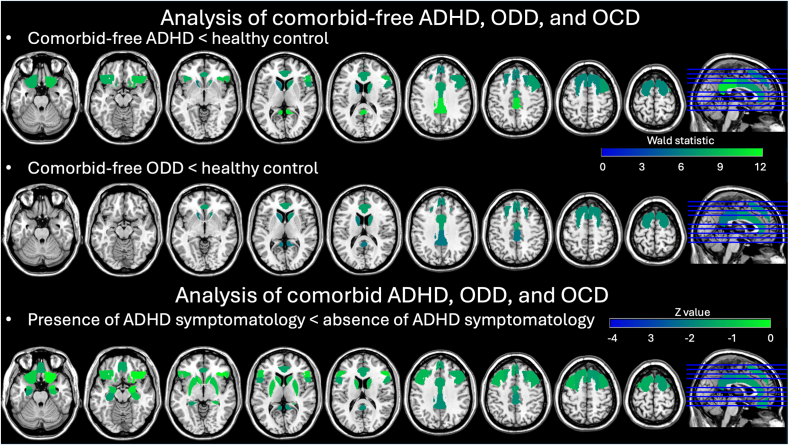
Significant Results of Hierarchical Models With Generalized Estimating Equations for Sample Entropy of Attention-Deficit/Hyperactivity Disorder (ADHD), Oppositional Defiant Disorder (ODD), and Obsessive-Compulsive Disorder (OCD) in the Comorbidity-Free and Comorbid Presentations ***Note:****All results present with* p *< .05 and Benjamini–Hochberg correction.*

For the ROI analysis, we found main effects of Group in the bilateral superior frontal gyrus, right middle frontal gyrus, right inferior frontal gyrus, and anterior cingulate gyrus (*χ*^2^= 10.177 to 13.261, *p* < .05, and BH corrected) in the cool EF network, and in the anterior cingulate gyrus, posterior cingulate gyrus, bilateral frontal orbital cortex, and bilateral caudate in the hot EF network (*χ*^2^= 10.138 to 15.476, *p* < .05, and BH corrected). The post hoc tests indicated that (1) the participants with comorbidity-free ADHD had lower SampEn in the bilateral superior frontal gyrus, right middle frontal gyrus, right inferior frontal gyrus, and anterior cingulate gyrus in the cool EF network (Wald statistic = 6.355 to 9.420, *p* < .05, and BH corrected) and in the anterior cingulate gyrus, posterior cingulate gyrus, bilateral frontal orbital cortex, and bilateral caudate in the hot EF network relative to the controls (Wald statistic = 5.913-10.798, *p* < .05, and BH corrected); (2) the participants with comorbidity-free ODD had lower SampEn in the bilateral superior frontal gyrus and anterior cingulate gyrus in the cool EF network (Wald statistic = 6.650-7.518, *p* < .05, and BH corrected) and in the anterior cingulate gyrus, posterior cingulate gyrus, and bilateral caudate in the hot EF network compared to the controls (Wald statistic = 5.682-6.873, *p* < .05, and BH corrected); (3) the participants with comorbidity-free OCD and controls did not have significantly different SampEn in any ROI of any EF network (Table 3 and Figure 2).

**Table 3:** Results of Hierarchical Models With Generalized Estimating Equations for Sample Entropy of Attention-Deficit/Hyperactivity Disorder (ADHD), Oppositional Defiant Disorder (ODD), and Obsessive-Compulsive Disorder (OCD) in the Comorbidity-Free and Comorbid Presentations

EF network	ROI	% Difference of expected SampEn for participants with comorbidity-free disease vs healthy controls			% Difference of expected SampEn in the presence of symptomatology vs absence of symptomatology		
		ADHD, %	ODD, %	OCD, %	ADHD, %	ODD, %	OCD, %
Cool	Right superior frontal gyrus	–10.0∗	–11.4∗	–1.8	–19.8∗	3.1	–16.7
	Left superior frontal gyrus	–9.9∗	–11.9∗	–2.0	–20.3∗	3.5	–16.8
	Right middle frontal gyrus	–9.8∗	–8.1	–1.7	–21.5∗	3.3	–18.1
	Left middle frontal gyrus	–8.1	–8.1	–2.1	–21.8∗	3.9	–17.9
	Right pars triangularis	–9.6∗	–6.0	–1.2	–23.5∗	2.9	–20.6
	Left pars triangularis	–6.7	–6.5	–1.0	–21.7∗	3.2	–18.5
	Right pars opercularis	–7.9∗	–6.7	–0.9	–18.9∗	2.6	–16.3
	Left pars opercularis	–6.6	–4.8	–0.8	–18.2∗	3.4	–14.8
	Anterior cingulate gyrus	–7.5∗	–8.6∗	–1.2	–14.3∗	2.9	–11.4
	Right hippocampus	–3.0	–3.2	0.6	–13.1∗	2.0	–11.1
	Left hippocampus	–3.7	–2.4	0.9	–9.6∗	2.0	–7.6
Hot	Frontal medial cortex	–6.2	–8.4	0.2	–21.0∗	3.9	–17.1
	Anterior cingulate gyrus	–7.5∗	–8.6∗	–1.2	–14.3∗	2.9	–11.4
	Posterior cingulate gyrus	–7.9∗	–6.4∗	0.2	–12.2∗	1.9	–10.3
	Right frontal orbital cortex	–7.9∗	–6.6	0	–22.4∗	2.9	–19.6
	Left frontal orbital cortex	–7.7∗	–5.9	–0.8	–19.9∗	3.5	–16.4
	Right caudate	–6.5∗	–6.9∗	0.8	–16.0∗	2.3	–13.7
	Left caudate	–5.7∗	–6.8∗	0.6	–16.7∗	2.6	–14.1
	Right putamen	–5.5	–4.3	0.5	–17.4∗	1.8	–15.5
	Left putamen	–5.6	–3.6	0	–16.1∗	2.3	–13.8
	Right amygdala	–4.0	–4.7	1.4	–17.6∗	2.3	–15.3
	Left amygdala	–5.0	–4.2	0.2	–13.7∗	2.6	–11.2
	Right accumbens	–3.9	–6.0	–0.3	–18.1∗	2.6	–15.5
	Left accumbens	–4.1	–6.9	0.5	–19.5∗	2.8	–16.7

Note: All results present with *p* < .05 and Benjamini–Hochberg correction. EF = executive function; ROI = region of interest; SampEn = sample entropy.

### Analysis for the Whole ADHD Sample

The whole ADHD sample showed significantly lower SampEn than the healthy controls in both EF networks (z = −2.881 and −3.268, *p* < .05, and BH corrected). In addition, the whole ADHD sample had significantly lower SampEn than the healthy controls in every ROI in each EF network (*z* = −3.617 to −2.550, *p* < .05, and BH corrected).

### Analysis for Comorbid ADHD, ODD, and OCD

On the network level, the presence of ADHD symptomatology was associated with significantly lower SampEn in both EF networks (z = −2.824 and −3.139, *p* < .05, and BH corrected). There was no significant difference between the presence and absence of ODD symptomatology and between the presence and absence of OCD symptomatology in each EF network.

For the ROI analysis, we found that the presence of ADHD symptomatology was associated with significantly lower SampEn in every ROI in each EF network (*z* = −3.973 to −2.235, *p* < .05, and BH corrected) (Table 3 and Figure 2). There was no significant difference between the presence and absence of ODD symptomatology and between the presence and absence of OCD symptomatology in any ROI.

### Secondary Analyses

#### Adding Sociodemographic Covariates

Adding sex, age, and YPDS as covariates in the hierarchical models with GEE did not result in conclusions different from those for our main analyses. Details are presented below.

In terms of the analysis for comorbidity-free ADHD, ODD, and OCD, we found significant main effects of Group in each network (*p* < .05 and BH corrected). Post hoc tests indicated that participants with comorbidity-free ADHD and comorbidity-free ODD had significantly lower SampEn than the controls in the cool EF network (Wald statistic = 7.146 and 5.254, *p* < .05, and BH corrected) and in the hot EF network (Wald statistic = 8.709 and 5.313, *p* < .05, and BH corrected). SampEn of participants with comorbidity-free OCD was not significantly different from that of the controls in either EF network. For the ROI analysis, we found main effects of Group in the bilateral superior frontal gyrus, right middle frontal gyrus, right inferior frontal gyrus, and anterior cingulate gyrus (*χ*^2^= 10.217-12.928, *p* < .05, and BH corrected) in the cool EF network, and in the anterior cingulate gyrus, posterior cingulate gyrus, bilateral frontal orbital cortex, and bilateral caudate in the hot EF network (*χ*^2^= 10.275-15.819, *p* < .05, and BH corrected). The post hoc tests indicated that (1) the participants with comorbidity-free ADHD had lower SampEn in the bilateral superior frontal gyrus, right middle frontal gyrus, right inferior frontal gyrus, and anterior cingulate gyrus in the cool EF network (Wald statistic = 6.661-9.718, *p* < .05, and BH corrected) and in the anterior cingulate gyrus, posterior cingulate gyrus, bilateral frontal orbital cortex, and bilateral caudate in the hot EF network relative to the controls (Wald statistic = 5.790-11.153, *p* < .05, and BH corrected); (2) the participants with comorbidity-free ODD had lower SampEn in the bilateral superior frontal gyrus and anterior cingulate gyrus in the cool EF network (Wald statistic = 6.523-6.996, *p* < .05, and BH corrected) and in the anterior cingulate gyrus, posterior cingulate gyrus, and bilateral caudate in the hot EF network compared to the controls (Wald statistic = 5.574 to 6.996, *p* < 0.05, and BH corrected); (3) the participants with comorbidity-free OCD and controls did not have significantly different SampEn in any ROI of any EF network (Table S2, available online).

In terms of the analysis for the whole ADHD sample, the whole ADHD sample showed significantly lower SampEn than the healthy controls in both EF networks (z = –2.879 and –3.273, *p* < .05, and BH corrected). In addition, the whole ADHD sample had significantly lower SampEn than the healthy controls in every ROI in each EF network (*z* = –3.651 to –2.525, *p* < .05, and BH corrected).

In terms of the analysis for comorbid ADHD, ODD, and OCD, the presence of ADHD symptomatology was associated with significantly lower SampEn in both EF networks (z = –2.710 and -2.959, *p* < 0.05, and BH corrected). There was no significant difference between the presence and absence of ODD symptomatology and between the presence and absence of OCD symptomatology in each EF network. For the ROI analysis, we found that the presence of ADHD symptomatology was associated with significantly lower SampEn in every ROI in each EF network (*z* = –3.743 to –2.120, *p* < .05, and BH corrected). There was no significant difference between the presence and absence of ODD symptomatology and between the presence and absence of OCD symptomatology in any ROI (Table S2, available online, provides more information).

### Splitting of the Other Comorbidities

We replicated the analysis for comorbid ADHD, ODD, and OCD by dividing X symptomatology into 3 categories: mood disorder symptomatology, anxiety symptomatology, and other symptomatology. The results showed that ADHD symptomatology was associated with significantly lower SampEn in both EF networks (z = –3.475 and –3.976, *p* < .05, BH corrected), whereas ODD symptomatology was associated with significantly higher SampEn in both EF networks (*z* = 2.139 and 2.177, *p* < .05, BH corrected). There was no significant difference between the presence and absence of OCD symptomatology in each EF network. For the ROI analysis, we found that the presence of ADHD symptomatology was associated with significantly lower SampEn in every ROI in each EF network (*z* = –4.413 to –3.105, *p* < .05, and BH corrected). There was no significant difference between the presence and absence of ODD symptomatology and between the presence and absence of OCD symptomatology in any ROI (Table S3, available online, provides more information).

#### Effects of Number of Comorbidities

Mean SampEn of each EF network was plotted against the number of comorbidities for each participant in Figure S2, available online. We found no significant association between SampEn and the number of comorbidities in any EF network and in any ROI of any EF network.

#### Association Between Complexity and Symptom Severity

No significant associations were found between SampEn and raw ADHD scores in any EF network or ROI for the whole ADHD sample. Similarly, no significant associations were observed between SampEn and raw ODD scores in the whole ODD sample, or between SampEn and raw OCD scores in the whole OCD sample.

#### Medication Effect

There was no significant difference in SampEn between the medicated and nonmedicated ADHD participants in any EF network or in any ROI.

## Discussion

We demonstrated that individuals with comorbidity-free ADHD exhibited significantly reduced SampEn across many of the ROIs in both cold and hot EF networks, whereas comorbidity-free ODD showed reduced SampEn in a subset of overlapping ROIs. When ADHD co-occurred with various comorbidities, we showed that the reduction in SampEn affected all ROIs within both EF networks, suggesting a cumulative disease burden and more severe deficits. Our analysis further revealed that reduced SampEn observed in the whole ADHD sample is driven primarily by the ADHD symptomatology rather than the severity of comorbidities.

Literature has indicated the comorbidity rate of ODD within children and adolescents with ADHD is around 26% and that the rate of co-occurrence of OCD within ADHD ranges from 3% to 7.5%.^4^^,^^5^ In the current ADHD sample, the probability of being diagnosed as ODD was 30%, consistent with published rates, whereas the rate of being diagnosed with OCD was 22.5%, notably higher than expected. Independent-sample *t* tests indicated that the ADHD score of the individuals with ADHD plus various comorbidities was significantly higher than in individuals with comorbidity-free ADHD (mean difference in ADHD score = 2.475, *t*_892_ = 6.514, *p* < .05); the ODD score of the individuals with ODD plus various comorbidities was significantly higher than in individuals with comorbidity-free ODD (mean difference in ODD score = 2.916, *t*_304_ = 8.060, *p* < .05); and the OCD score of the individuals with OCD plus various comorbidities was significantly higher than in individuals with comorbidity-free OCD (mean difference in OCD score = 3.149, *t*_247_) = 7.317, *p* < .05). The current analysis suggested that symptom severity was greater when it co-occurred with other symptoms compared to when it presented alone, which was consistent with observations in the literature.^43^, ^44^, ^45^

Previous studies have reported reduced rsfMRI complexity in ADHD,^29^, ^30^, ^31^ which was corroborated by our findings using a larger sample and accounting for comorbidities. Specifically, we observed reduced SampEn in individuals ADHD with comorbid pathologies as well as in individuals with comorbidity-free ADHD within both cool and hot EF networks. Existing literature on task-based fMRI studies showed that ADHD is characterized predominantly by functional alterations in the cool EF system,^8^ whereas previous rsfMRI-based meta-analyses reported that individuals with ADHD had altered functional connectivity between regions in both cool and hot EF networks.^46^, ^47^, ^48^ Notably, in our previous study,^31^ we reported decreased complexity and altered functional connectivity in the same cohort of children with comorbidity-free ADHD but compared to a completely distinct set of matched controls, thus providing evidence of reliability of the current finding and a relation between complexity and connectivity in brain networks.

In individuals with comorbidity-free ODD, our study revealed reduced SampEn within both EF networks, although fewer regions were affected compared to those in comorbidity-free ADHD. ODD has primarily been associated with abnormalities in the hot EF system, and most functional imaging studies have focused on this EF system.^7^ There is sparse evidence for cold EF deficits.^49^ Our study is the first to investigate the complexity of brain function in ODD and provides supportive evidence for impairments not only in the hot EF network but also in the cold EF network. When ODD was comorbid with ADHD, it enhanced the complexity of both networks as shown by hierarchical GEE models, in which X symptomatology was divided into 3 categories of mood disorder, anxiety, and other symptomatologies. This pattern suggests that the neural alterations associated with ODD may differ depending on whether it presents alone or in combination with ADHD, highlighting the importance of considering comorbidity when interpreting the neural underpinnings of ODD.

Research on OCD complexity has so far exclusively focused on adult populations, with studies reporting both increased and decreased complexity.^32^, ^33^, ^34^ Similarly, structural and functional neuroimaging findings in adult populations with OCD have revealed varied findings that lack generalizability.^50^ Neuroimaging in pediatric OCD is a growing field, and findings across studies and modalities vary, with most commonly observed abnormalities in cortico-striatal loops.^51^ Although our study is the first to investigate the complexity of pediatric OCD, we did not find significantly different SampEn in the individuals with comorbidity-free OCD compared to the controls in the EF networks. This, however, does not preclude differences on an individual level or other brain networks. Although pediatric OCD is often considered a chronic condition, longitudinal studies suggest that the course can vary considerably. For example, Bloch *et al.*^52^ found that 20 of 44 children with OCD experienced significant symptom improvement or remission by early adulthood. It underscored the heterogeneity in long-term outcomes for pediatric OCD, thus warranting further research to elucidate the neurophysiology and developmental trajectories of OCD.

Our analysis of the entire ADHD sample suggests more widespread deficits, including all ROIs within both EF networks, when comorbidities are present. However, there was no significant association between SampEn and the number of comorbidities. When the whole ADHD sample was divided into subgroups with varying comorbidities (ODD, OCD, and other psychiatric disorders), ADHD symptomatology remained the dominant factor in reducing SampEn in all ROIs. In contrast, the symptomatologies of ODD and OCD did not significantly contribute to SampEn alterations in these ROIs. Hence, reduced SampEn observed in the whole ADHD sample is driven primarily by ADHD symptomatology rather than the severity of comorbidities. So far, only 2 fMRI studies have compared ADHD comorbid with ODD with comorbidity-free ADHD, and findings indicated that the comorbid ADHD was associated with greater impairment in brain function than ADHD alone in the regions associated with EF, which aligned with the current observation. Noordemeer^53^ reported that ADHD comorbid with ODD revealed reduced activity in the paracingulate and superior frontal gyrus during failed inhibition in the inhibition task. Wang *et al.*^54^ reported enhanced resting-state functional connectivity in ADHD comorbid with ODD in the regions including the superior/middle/medial frontal gyrus and middle cingulate gyrus using the bilateral anterior cerebellum as the seed.

Several caveats should be considered with respect to the current results. First, in our analysis of comorbid ADHD, ODD, and OCD, the model assumed that SampEn depended on a linear combination of ADHD, ODD, OCD, and other symptomatologies but not their interactions. To build a feasible model that incorporates interaction terms, it would be necessary to include individuals with ODD and/or OCD but without ADHD. In the current study, we focused on the changes in complexity when ADHD coexisted with ODD, OCD, and other conditions. Including participants without ADHD but with other psychiatric diagnoses would deviate from this objective, so we opted for a simpler model without interaction terms. Second, Sokunbi *et al.*^30^ reported a negative association between SampEn and the ADHD score in multiple regions, including the medial frontal gyrus. The current study did not find a significant association between complexity and ADHD, ODD, and OCD symptom severity. Third, we observed no significant differences in SampEn between medicated and nonmedicated ADHD preadolescents, suggesting that stimulant treatment status may not have a substantial impact on functional complexity at this developmental stage. Fourth, because of an anomaly within the ABCD Study infrastructure, the ADHD diagnosis reported in Release 3.0 was later determined to be invalid, and consequently, the diagnosis was removed in Release 4.0. The diagnosis of ODD in Release 5.1 permits either current or past symptoms to count toward a current diagnosis; this should be updated so that only current symptoms contribute. Applying the updated ODD criteria would likely reduce the number of participants classified with ODD. Similarly, adopting the new ADHD criteria would substantially decrease our ADHD sample size. As a result, the ADHD + ODD and ADHD + ODD + OCD groups could potentially drop to zero participants, making hierarchical models with GEE infeasible. In light of these considerations, we chose to retain our current approach based on the information available in Release 3.0. With regard to the estimation of complexity, we based our analysis on the raw rsfMRI data and not on any preprocessed or cleaned version distributed by the ABCD Consortium. Therefore, with regard to the individual participants’ imaging metrics, the release version had no effect on the results. Fifth, our ROIs were formed based on the Harvard–Oxford atlas. The Harvard–Oxford atlas offers robust anatomical coverage and is widely implemented in major neuroimaging toolboxes such as FSL and CONN, facilitating standardization, replicability, and generalizability of our findings. Although functionally derived atlases (eg, Yeo or Power) are valuable for exploring whole-brain connectivity patterns, they are less suitable when specific anatomically grounded hypotheses are being tested, as in our case. In addition, structural atlases such as the Harvard–Oxford atlas do not preclude meaningful functional interpretations; they serve as a reliable framework for summarizing regional activity and complexity in a reproducible way, especially when functional hypotheses are anchored in anatomical models. Thus, the use of the Harvard–Oxford atlas in this resting-state study was both methodologically sound and theoretically aligned with our study objectives. Sixth, although the groups of ADHD + OCD and ADHD + ODD + OCD had very small sample sizes, the current results were not largely driven by them. Our analyses did not rely on stratified subgroup comparisons. Instead, we modeled the presence or absence of each symptom domain (ADHD, ODD, OCD) as independent variables across the entire sample, which allowed us to pool data and to improve statistical power. This approach minimizes the risk that results are driven solely by small subgroups. We encourage future studies with larger comorbid samples to replicate and extend these findings. Seventh, our sample consisted of children 9 to 10 years of age, a developmental period that precedes the typical onset of many mood and anxiety disorders, which more commonly emerge in mid- to late adolescence. Although these conditions are known to affect executive functioning, their impact is less likely to be evident in the current age range. In the current whole ADHD sample (n = 1,085), only 13 children met criteria for major depressive disorder, 66 for generalized anxiety disorder, and 6 for both. Because of these small sample sizes, we were unable to perform the GEE analyses to examine the effects of MDD and GAD symptoms on functional complexity. Future research with older samples or longitudinal designs will be critical to assess how the emergence of mood and anxiety disorders may influence brain function and executive networks over time. Eighth, although our analyses focused on hot and cold EF networks and their ROIs separately, we acknowledge that these systems are highly interconnected. Treating them as distinct may risk overlooking the functional interplay between them, as deficits in one network or ROI could exacerbate dysfunctions in the other. By definition, traditional fMRI functional connectivity and fMRI-based complexity measures such as SampEn can be viewed as complementary indices of network function. Whereas functional connectivity quantifies the coherence of signals and thus communication between brain areas, SampEn provides an index of a region’s intrinsic signal richness. Accordingly, functional connectivity does not provide information about a region in isolation, whereas SampEn does not directly capture interrelations between areas. Importantly, however, the complexity of a brain region is shaped by its interactions with other areas, and disturbances in local signal processing can, in turn, influence estimated connectivity. Our findings on SampEn offer an important first step in characterizing the intrinsic complexity of each ROI and each network; however, future work integrating entropy-based measures with connectivity analyses will be critical for capturing both within-network/ROI dynamics and cross-network/ROI interactions. Such approaches may yield a more comprehensive understanding of the neural underpinnings of ADHD, ODD, and OCD.

In summary, our study highlights the importance of accounting for comorbid status when examining functional brain alterations in youth with neuropsychiatric conditions. We found overlapping reductions in neural complexity within executive function (EF) networks in comorbidity-free ADHD and ODD, with ADHD showing more extensive effects. In comorbid presentations, SampEn reductions were even more pronounced, but our results suggest that these reductions were driven primarily by ADHD symptomatology rather than by symptoms of co-occurring conditions. Notably, ODD symptoms did not have a further impact on SampEn in any ROI when co-occurring with ADHD. These findings underscore that ADHD plays a central role in shaping functional brain alterations, even in the presence of other diagnoses. Clinically, this suggests that for youth with comorbid ADHD, interventions may be most effective when they prioritize the treatment of ADHD symptoms. Furthermore, SampEn may offer a promising neurobiological marker for capturing ADHD-related alterations in brain complexity, supporting its potential utility in refining diagnoses and guiding individualized treatment strategies.

## CRediT authorship contribution statement

**Ru Zhang:** Writing – review & editing, Writing – original draft, Visualization, Validation, Methodology, Investigation, Formal analysis, Data curation, Conceptualization. **Steven Cen:** Writing – review & editing, Methodology, Investigation, Formal analysis. **Dilmini Wijesinghe:** Writing – review & editing, Methodology, Investigation. **Leon Aksman:** Writing – review & editing, Methodology, Investigation, Formal analysis, Conceptualization. **Stuart B. Murray:** Writing – review & editing, Resources, Methodology, Investigation, Formal analysis, Conceptualization. **Christina J. Duval:** Methodology. **Danny J.J. Wang:** Writing – review & editing, Supervision, Resources, Investigation, Funding acquisition, Conceptualization. **Kay Jann:** Writing – review & editing, Supervision, Resources, Methodology, Investigation, Funding acquisition, Conceptualization.
