# Supplementary material for: Elucidating Distinct and Common fMRI-Complexity Patterns in Preadolescent Children With Attention-Deficit/Hyperactivity Disorder, Oppositional Defiant Disorder, and Obsessive-Compulsive Disorder
Source: JAACAP Open. 2025 Nov 27;4(2):254–67. doi: 10.1016/j.jaacop.2025.11.008 (PMC13043473; doi:10.1016/j.jaacop.2025.11.008)
Supplement: Supplemental Material [file mmc1.docx]

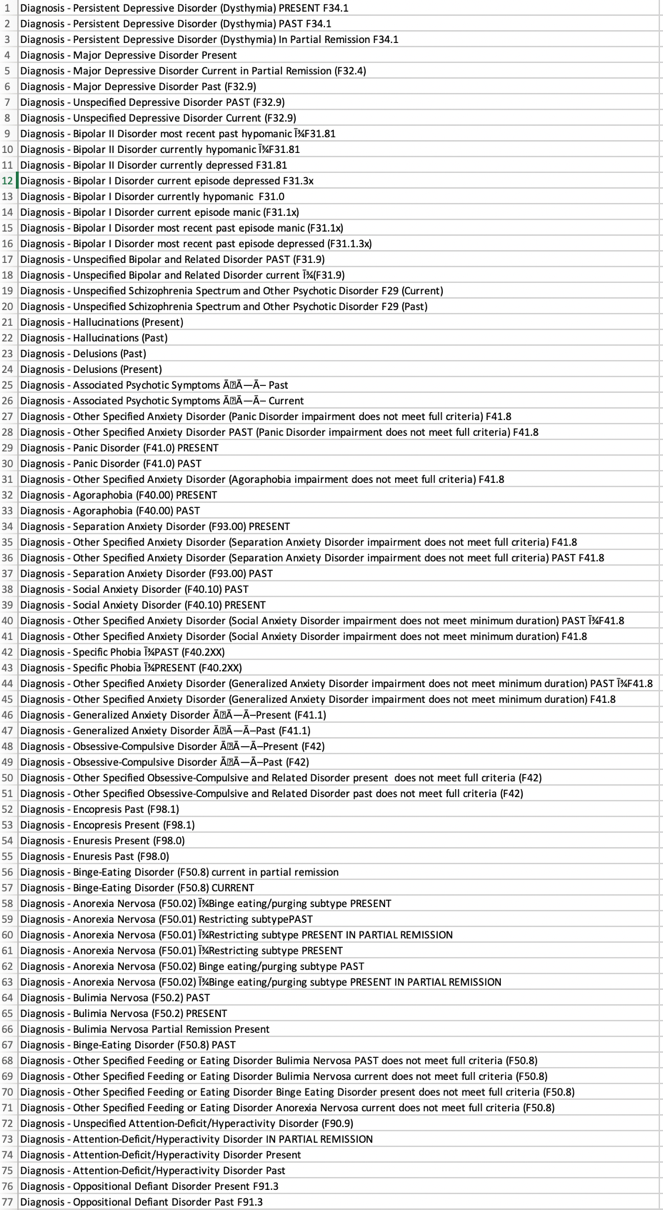

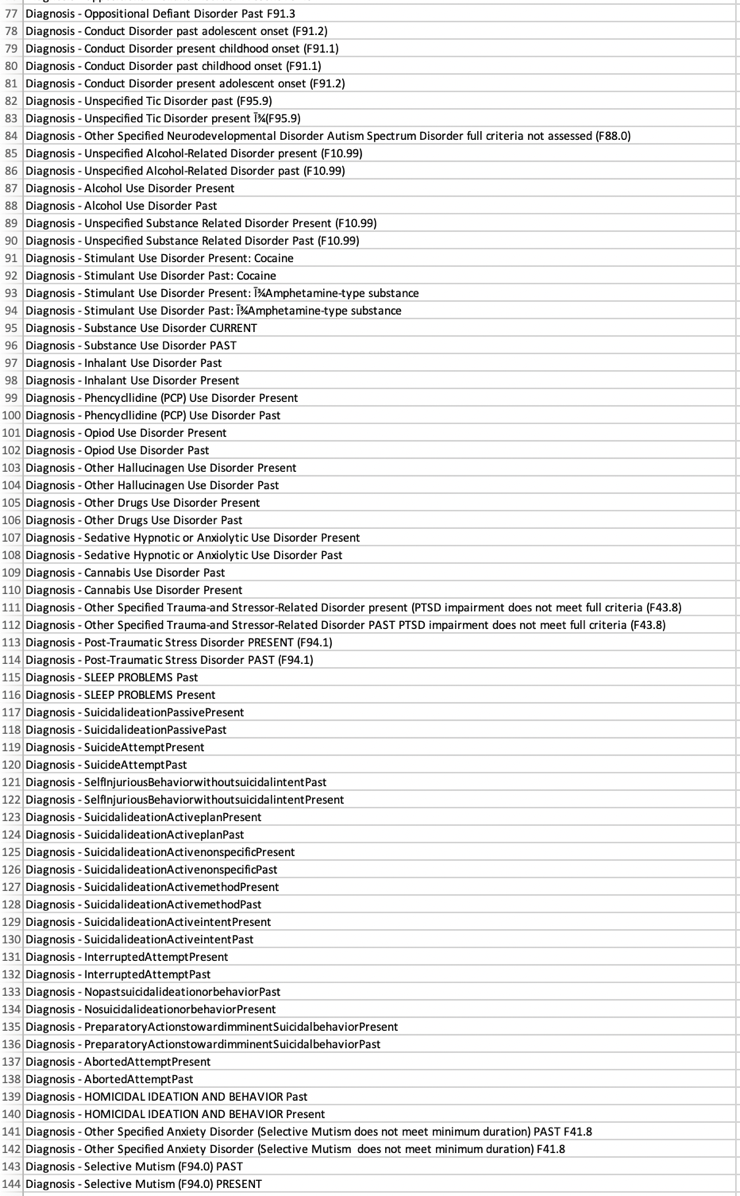


Figure S1. 144 psychiatric diagnoses evaluated by the ABCD study.

Supplement 1

Mood disorder symptomatology was defined present if one or more of the following diagnoses were made:

| Diagnosis - Persistent Depressive Disorder (Dysthymia) PRESENT F34.1 |
| --- |
| Diagnosis - Persistent Depressive Disorder (Dysthymia) PAST F34.1 |
| Diagnosis - Persistent Depressive Disorder (Dysthymia) In Partial Remission F34.1 |
| Diagnosis - Major Depressive Disorder Present |
| Diagnosis - Major Depressive Disorder Current in Partial Remission (F32.4) |
| Diagnosis - Major Depressive Disorder Past (F32.9) |
| Diagnosis - Unspecified Depressive Disorder PAST (F32.9) |
| Diagnosis - Unspecified Depressive Disorder Current (F32.9) |
| Diagnosis - Bipolar II Disorder most recent past hypomanic F31.81 |
| Diagnosis - Bipolar II Disorder currently hypomanic F31.81 |
| Diagnosis - Bipolar II Disorder currently depressed F31.81 |
| Diagnosis - Bipolar I Disorder current episode depressed F31.3x |
| Diagnosis - Bipolar I Disorder currently hypomanic F31.0 |
| Diagnosis - Bipolar I Disorder current episode manic (F31.1x) |
| Diagnosis - Bipolar I Disorder most recent past episode manic (F31.1x) |
| Diagnosis - Bipolar I Disorder most recent past episode depressed (F31.1.3x) |
| Diagnosis - Unspecified Bipolar and Related Disorder PAST (F31.9) |
| Diagnosis - Unspecified Bipolar and Related Disorder current (F31.9) |

Anxiety symptomatology was defined present if one or more of the following diagnoses were made:

| Diagnosis - Other Specified Anxiety Disorder (Panic Disorder impairment does not meet full criteria) F41.8 |
| --- |
| Diagnosis - Other Specified Anxiety Disorder PAST (Panic Disorder impairment does not meet full criteria) F41.8 |
| Diagnosis - Panic Disorder (F41.0) PRESENT |
| Diagnosis - Panic Disorder (F41.0) PAST |
| Diagnosis - Other Specified Anxiety Disorder (Agoraphobia impairment does not meet full criteria) F41.8 |
| Diagnosis - Agoraphobia (F40.00) PRESENT |
| Diagnosis - Agoraphobia (F40.00) PAST |
| Diagnosis - Separation Anxiety Disorder (F93.00) PRESENT |
| Diagnosis - Other Specified Anxiety Disorder (Separation Anxiety Disorder impairment does not meet full criteria) F41.8 |
| Diagnosis - Other Specified Anxiety Disorder (Separation Anxiety Disorder impairment does not meet full criteria) PAST F41.8 |
| Diagnosis - Separation Anxiety Disorder (F93.00) PAST |
| Diagnosis - Social Anxiety Disorder (F40.10) PAST |
| Diagnosis - Social Anxiety Disorder (F40.10) PRESENT |
| Diagnosis - Other Specified Anxiety Disorder (Social Anxiety Disorder impairment does not meet minimum duration) PAST F41.8 |
| Diagnosis - Other Specified Anxiety Disorder (Social Anxiety Disorder impairment does not meet minimum duration) F41.8 |
| Diagnosis - Specific Phobia PAST (F40.2XX) |
| Diagnosis - Specific Phobia PRESENT (F40.2XX) |
| Diagnosis - Other Specified Anxiety Disorder (Generalized Anxiety Disorder impairment does not meet minimum duration) PAST F41.8 |
| Diagnosis - Other Specified Anxiety Disorder (Generalized Anxiety Disorder impairment does not meet minimum duration) F41.8 |
|  |
| Diagnosis - Generalized Anxiety Disorder Past (F41.1)  Diagnosis - Other Specified Anxiety Disorder (Selective Mutism does not meet minimum duration) PAST F41.8  Diagnosis - Other Specified Anxiety Disorder (Selective Mutism does not meet minimum duration) F41.8 |


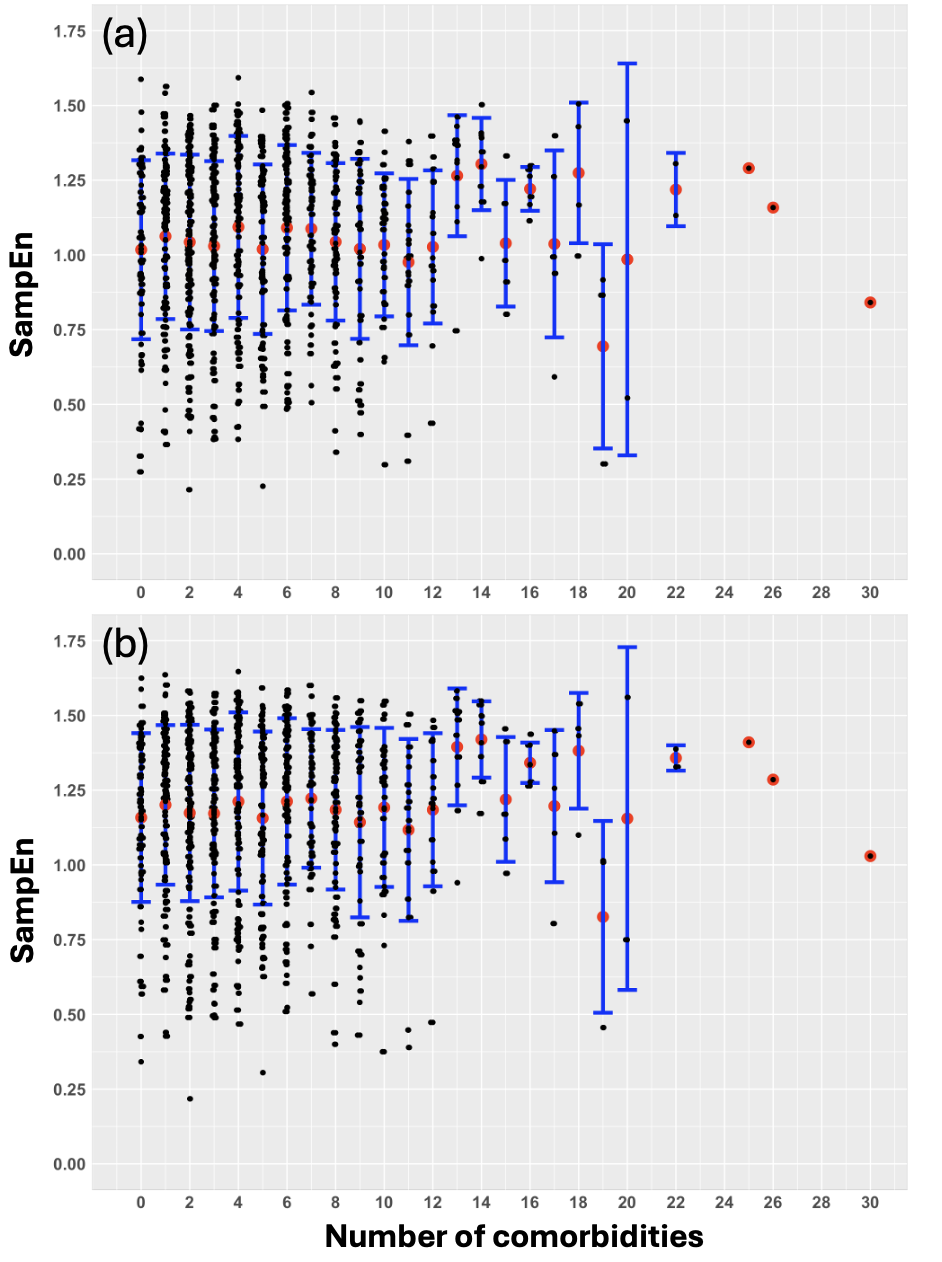


Figure S2. Mean SampEn Plotted Against the Number of Comorbidities for Each Executive Function Network. (a) Mean SampEn of the cool EF network for each ADHD participant, plotted against the number of comorbidities. The red dot and blue line segment display the mean and standard deviation of each condition. (b) Mean SampEn of the hot EF network for each ADHD participant, plotted against the number of comorbidities. The red dot and blue line segment display the mean and standard deviation of each condition. Note: Comorbidities here referred to psychiatric comorbidities, which included 143 possible diagnoses, as shown in Figure S1. Some participants received multiple diagnostic codes within a category (e.g., separation anxiety, social anxiety, generalized anxiety), which could lead to high comorbidity counts. Although the numbers might appear high, they reflected the comprehensive scope of diagnostic coding rather than approximately 25 entirely distinct disorders. ADHD = attention-deficit/hyperactivity disorder; EF = executive function; SampEn = sample entropy.

Table S1. A list of prescriptions for attention-deficit/hyperactivity disorder.

| Prescription |
| --- |
| Adderall |
| Azstarys |
| Serdexmethylphenidate |
| Dexmethylphendiate |
| Concerta |
| Methylphenidate |
| Focalin |
| Intuniv |
| Guanfacine |
| Gelbree |
| Viloxazine |
| Ritalin |
| Strattera |
| Atomoxetine |
| Vyvanse |
| Adzenys |
| Evekeo |
| Daytrana |
| Quillivant |
| Amphetamine |
| Clonidine |
| Metadate |

Table S2. The results of hierarchical generalized estimating equations with sociodemographic covariates for sample entropy of attention-deficit/hyperactivity disorder, oppositional defiant disorder, and obsessive-compulsive disorder in the comorbid-free and comorbid presentations.

| EF network | ROI | Percent difference of expected SampEn for comorbid-free disease vs. healthy control | | | Percent difference of expected SampEn in presence of symptomatology vs. absence of symptomatology | | |
| --- | --- | --- | --- | --- | --- | --- | --- |
|  |  | ADHD | ODD | OCD | ADHD | ODD | OCD |
| Cool | Right superior frontal gyrus | -10.2%* | -11.5%* | -1.5% | -19.7%* | 3.0% | -16.6% |
|  | Left superior frontal gyrus | -10.1%* | -12.1%* | -1.7% | -20.2%* | 3.5% | -16.7% |
|  | Right middle frontal gyrus | -9.9%* | -8.1% | -1.5% | -21.3%* | 3.3% | -18.0% |
|  | Left middle frontal gyrus | -8.3% | -8.3% | -2.0% | -21.6%* | 3.9% | -17.8% |
|  | Right pars triangularis | -9.7%* | -6.1% | -1.0% | -23.2%* | 9.0% | -20.4% |
|  | Left pars triangularis | -6.8% | -6.7% | -1.1% | -21.4%* | 6.0% | -18.3% |
|  | Right pars opercularis | -8.0%* | -6.6% | -0.7% | -18.6%* | 12.5% | -16.2% |
|  | Left pars opercularis | -6.7% | -5.1% | 0.9% | -18.0%* | 3.9% | -14.6% |
|  | Anterior cingulate gyrus | -7.5%* | -8.5%* | -1.1% | -13.9%* | 2.7% | -11.2% |
|  | Right hippocampus | -3.0% | -3.2% | 0.5% | -12.9%* | 1.9% | -10.9% |
|  | Left hippocampus | -3.8% | -2.4% | 0.9% | -9.4%* | 1.9% | -7.4% |
| Hot | Frontal medial cortex | -6.3% | -8.4% | 0.3% | -20.7%* | 3.9% | -16.8% |
|  | Anterior cingulate gyrus | -7.5%* | -8.5%* | -1.1% | -13.9%* | 2.7% | -11.2% |
|  | Posterior cingulate gyrus | -8.0%* | -6.5%* | 0.2% | -11.9%* | 1.8% | -10.1% |
|  | Right frontal orbital cortex | -7.8%* | -6.6% | 0 | -22.0%* | 2.7% | -19.3% |
|  | Left frontal orbital cortex | -7.7%* | -5.9% | -0.7% | -19.5%* | 3.4% | -16.1% |
|  | Right caudate | -6.5%* | -6.9%* | 0.8% | -15.6%* | 2.2% | -13.5% |
|  | Left caudate | -5.7%* | -6.7%* | 0.7% | -16.4%* | 2.5% | -13.9% |
|  | Right putamen | -5.5% | -4.4% | 0.5% | -17.0%* | 1.7% | -15.3% |
|  | Left putamen | -5.6% | -3.6% | 0 | -15.9%* | 2.2% | -13.6% |
|  | Right amygdala | -4.0% | -4.7% | 1.4% | -17.3%* | 2.2% | -15.1% |
|  | Left amygdala | -5.1% | -4.2% | 0.2% | -13.5%* | 2.5% | -11.0% |
|  | Right accumbens | -3.9% | -6.0% | -0.4% | -17.7%* | 2.5% | -15.2% |
|  | Left accumbens | -4.0% | -7.0% | 0.5% | -19.1%* | 2.7% | -16.4% |

Note: All the results present with *p* < 0.05 and BH correction. ADHD = attention-deficit/hyperactivity disorder; EF = executive function; OCD = obsessive-compulsive disorder; ODD = oppositional defiant disorder; ROI = region of interest; SampEn = sample entropy.

Table S3. The results of hierarchical generalized estimating equations with covariates of mood disorder, anxiety, and other symptomatologies for sample entropy of attention-deficit/hyperactivity disorder, oppositional defiant disorder, and obsessive-compulsive disorder symptomatologies in the comorbid presentations.

| EF network | ROI | Percent difference of expected SampEn in presence of symptomatology vs. absence of symptomatology | | |
| --- | --- | --- | --- | --- |
|  |  | ADHD | ODD | OCD |
| Cool | Right superior frontal gyrus | -8.5%* | 3.6% | -4.8% |
|  | Left superior frontal gyrus | -8.6%* | 4.2% | -4.4% |
|  | Right middle frontal gyrus | -8.4%* | 3.7% | -4.8% |
|  | Left middle frontal gyrus | -7.8%* | 4.3% | -3.4% |
|  | Right pars triangularis | -9.2%* | 3.4% | -5.8% |
|  | Left pars triangularis | -8.9%* | 3.4% | -5.5% |
|  | Right pars opercularis | -7.6%* | 2.8% | -4.8% |
|  | Left pars opercularis | -8.0%* | 3.7% | -4.2% |
|  | Anterior cingulate gyrus | -6.8%* | 3.5% | -3.3% |
|  | Right hippocampus | -4.4%* | 2.0% | -2.4% |
|  | Left hippocampus | -4.5%* | 2.1% | -2.4% |
| Hot | Frontal medial cortex | -8.8%* | 4.3% | -4.5% |
|  | Anterior cingulate gyrus | -6.8%* | 3.5% | -3.3% |
|  | Posterior cingulate gyrus | -5.4%* | 2.1% | -3.3% |
|  | Right frontal orbital cortex | -8.8%* | 3.1% | -5.6% |
|  | Left frontal orbital cortex | -9.2%* | 3.9% | -5.3% |
|  | Right caudate | -6.3%* | 2.6% | -3.7% |
|  | Left caudate | -6.1%* | 3.0% | -3.1% |
|  | Right putamen | -6.0%* | 2.0% | -4.1% |
|  | Left putamen | -6.4%* | 2.8% | -3.6% |
|  | Right amygdala | -5.8%* | 2.3% | -3.5% |
|  | Left amygdala | -6.6%* | 2.8% | -3.8% |
|  | Right accumbens | -6.2%* | 2.9% | -3.3% |
|  | Left accumbens | -6.4%* | 3.1% | -3.4% |

Note: All the results present with *p* < 0.05 and BH correction. ADHD = attention-deficit/hyperactivity disorder; EF = executive function; OCD = obsessive-compulsive disorder; ODD = oppositional defiant disorder; ROI = region of interest; SampEn = sample entropy.
